# EU-TIRADS-Based Omission of Fine-Needle Aspiration and Cytology from Thyroid Nodules Overlooks a Substantial Number of Follicular Thyroid Cancers

**DOI:** 10.1155/2021/9924041

**Published:** 2021-09-27

**Authors:** Tamas Solymosi, Laszlo Hegedüs, Miklos Bodor, Endre V. Nagy

**Affiliations:** ^1^Endocrinology and Metabolism Clinic, Bugat Hospital, 20 Dozsa u, H-3200 Gyöngyös, Hungary; ^2^Department of Endocrinology, Kløvervænget 6, 5.sal, Odense University Hospital, DK-5000 Odense, Denmark; ^3^Division of Endocrinology, Department of Medicine, Faculty of Medicine, University of Debrecen, 98 Nagyerdei krt, H-4032 Debrecen, Hungary

## Abstract

**Background:**

The classification of nodules by Thyroid Imaging Reporting and Data Systems (TIRADS) is important in guiding management. Whether sensitivity in identifying thyroid cancers varies with thyroid cancer phenotype remains unclarified.

**Methods:**

The ultrasound (US) characteristics of nodules of 26,908 nodular goiter patients were recorded. Fine-needle aspiration cytology (FNA) was performed in all nodules >1 cm irrespective of US findings (*n* = 25,025) and in nodules between 5 mm and 10 mm with suspicious US characteristics (*n* = 1,883). Of the 3281 operated cases, 221, 30, and 23 were papillary (PTC), follicular (FTC), and medullary (MTC) cancers, respectively. The US-based indication of FNA, as defined by EU-TIRADS scores, combined with lesion size, was calculated. This study design is unique in avoiding the common selection bias when TIRADS' sensitivity is tested in a cohort selected for FNA and surgery based on the same US characteristics on which TIRADS is based.

**Results:**

The EU-TIRADS score influences decision of FNA in the 10–20 mm range. In such nodules (*n* = 118), the number of suspicious features (marked hypoechogenicity, microcalcifications, irregular shape, and irregular border) per lesion was lower in FTC (0.7 ± 0.6) than in PTC (1.7 ± 1.0) or MTC (1.8 ± 0.7; *p* < 0.02), resulting in EU-TIRADS scores of 4.1 ± 0.6, 4.8 ± 0.3, and 4.9 ± 0.2, respectively (*p* < 0.01). The EU-TIRADS-based FNA indication rate was lower in FTC (55.5%) compared to PTC (85.0%) and MTC (88.9%) (*p*=0.02).

**Conclusions:**

EU-TIRADS-defined suspicious US features are less common in FTC than in PTC and MTC. Therefore, a substantial number of FTCs in the 10–20 mm range escape surgery.

## 1. Introduction

There has been a continuous development over the past 40 years in the selection of thyroid nodules that qualify for fine-needle aspiration cytology (FNA). The main initial driver is used for recognizing all malignancies; the principle of reaching the highest sensitivity was supported by the introduction of thyroid ultrasound (US) in everyday practice. From the pre-US era, when FNA was indicated in palpable “cold” thyroid nodules [1], the number of biopsies gradually rose and peaked around the publication of the AACE/ACE guideline in 2006 (AACE-2006). This guideline suggested FNA of all nodules larger than 10 mm and even of smaller ones presenting clinical or US suspicion of cancer [2].

In the past 10 years, focus has changed and has mainly been driven by the principle of balancing diagnostic sensitivity and specificity and reducing the number of FNAs. The main tool for this change has been the consideration of suspicious ultrasound characteristics for the decision of performing FNA [3–11]. Practically, all guidelines published since 2006 link indications for cytology to suspicious US signs. There are some differences between various thyroid nodule image reporting and data systems (TIRADS) [5–9] in what is considered suspicious and in the smallest thyroid nodule size worth considering for cytology. Using the same approach, the various TIRADS have clarified which ultrasound patterns merit cytology; furthermore, sampling is not recommended for isoechoic nodules between 1 cm and 2 cm in diameter and for those between 1 cm and 1.5 cm that do not show suspicious signs, as stated by the AACE-TIRADS, ACR-TIRADS, EU-TIRADS [5, 6, 8], and ATA-TIRADS and KSTR-TIRADS [7, 9], respectively.

The bases of all TIRADS systems are those US characteristics which have diagnostic importance in the recognition of papillary thyroid carcinomas (PTC) [12]. The US characteristics of FTC differ profoundly from PTC characteristics [13–15]. Only a limited number of studies deal with the importance of TIRADS in medullary thyroid cancer (MTC) [16–20] and follicular thyroid cancer (FTC) [21–23]. To the best of our knowledge, there has been only one study which focuses on the diagnostic performance of TIRADS in patient selection for FNA in FTC [23]. The fact that TIRADS' performance has been tested almost exclusively in PTC patients supports the view that the clinical validity of TIRADS systems cannot be unconditionally extended to other forms of thyroid malignancy [24]. Therefore, while the US-centered diagnostic work-up can effectively identify PTCs, it is not known if TIRADS systems are similarly successful in finding FTCs and MTCs burdened by substantially higher risk of mortality [25–27].

In the present study, the effectiveness of EU-TIRADS-based nodule selection in the detection of FTC and MTC was retrospectively analyzed in a patient cohort which underwent FNA irrespective of the EU-TIRADS-based criteria for suspicion.

### 1.1. Patients and Methods

Between January 2006 and December 2018, 26 908 patients, evaluated at the Thyroid Clinic of the Bugat Pal Hospital (Gyöngyös, Hungary), were diagnosed with thyroid nodules. In all patients, US was performed and TSH was measured, supplemented with FT4 and FT3 if needed. The decision of FNA was made in accordance with the AACE-2006 guideline [2], i.e., US-guided FNA was performed in patients with nontoxic nodules larger than 10 mm in maximal diameter. The same protocol was followed throughout the 13 years, i.e., no alteration was made after the publication of the AACE/ACE/AME 2016 guidelines [5]. If the clinical examination, patient history, or US signs suggested increased risk of thyroid cancer, hypoechogenic nodules between 5 mm and 10 mm were also aspirated. FNA was also performed if the only suspicious sign was the presence of pathological cervical lymph nodes by US. For nonoperated patients, follow-up US was performed after 1–3 years, and FNA repeated if the nodule volume grew by more than 33%. US and cytology were performed by the same examiner (TS) with 22 years of experience in this field. In the first 6 years of the study, a SonoSite Micromaxx (Bothell, WA, USA) with a 5–10 MHz probe was used, while in the subsequent years, a Philips CX50 ultrasound machine (Eindhoven, the Netherlands) with a 5–12 Hz transducer was used for US of the thyroid and the neck.

The following standard US features of the nodules were recorded and analyzed: the three diameters, composition, echogenicity, presence of microcalcifications, taller-than-wide shape, and macrocalcifications (including peripheral eggshell calcification). Composition was classified as solid (solid portion ≥90%), predominantly solid (solid portion 50–90%), predominantly cystic (solid portion <50%), and cystic (solid portion <10%). The nodule was classified as hyperechoic, isoechoic, or hypoechoic compared to the surrounding nonnodular thyroid tissue. Hypoechoic nodules were subdivided as minimally/moderately hypoechogenic or markedly hypoechogenic, i.e., less or more hypoechoic as compared to the adjacent cervical muscles, respectively. Margins were classified as regular or irregular (lobulated or spiculated).

Altogether, 3281 patients were operated on. The indications for surgery were (i) suspicious cytology (Table 1), (ii) benign or repeatedly nondiagnostic cytology combined with clinical and/or US suspicion of malignancy, (iii) compression signs and/or symptoms, or (iv) patient preference. TNM and clinical staging were according to the most recent classification [28].

Based on final histology, 338 patients had thyroid cancer. After exclusion of patients with cancers other than PTC, FTC, or MTC, those who had previous thyroid surgery, and the 5 papillary cancers (because it was equivocal which of the nodules were malignant), 274 nodules remained and were retrospectively analyzed. Of these 274 nodules, 221 were PTC, 30 FTC, and 23 MTC. In multifocal carcinomas, only one focus (the largest one) was included in the analysis. The most important clinical data of the patients and the results of FNA at the first examination are summarized in Table 1.

EU-TIRADS scores were retrospectively generated in all 274 cases. The score for a given nodule was translated into “for” or “against” the subsequent use of FNA [8]. FNA was considered indicated even if it was substantiated merely by the presence of pathological lymph nodes.

The EU-TIRADS score, based on US characteristics other than the maximal diameter of the nodule, influences the decision of FNA in lesions between 10 mm and 20 mm in maximal diameter. Nodules with their largest diameter in this range, and proven to be PTC, FTC, or MTC by final histology, were retrospectively analyzed regarding the prevalence of suspicious US characteristics: marked hypoechogenicity, taller-than-wide shape, irregular (lobulated or spiculated) margins, and microcalcifications [8].

The retrospective analysis of the data stored in the hospital database has been preapproved by the Ethics Committee of the Bugat Pal Hospital, Gyöngyös, Hungary. For statistical comparisons, Fisher's exact test and the Mann–Whitney *U* test were used.

## 2. Results

### 2.1. Size Distribution of the Three Cancer Types

**Figure 1:**
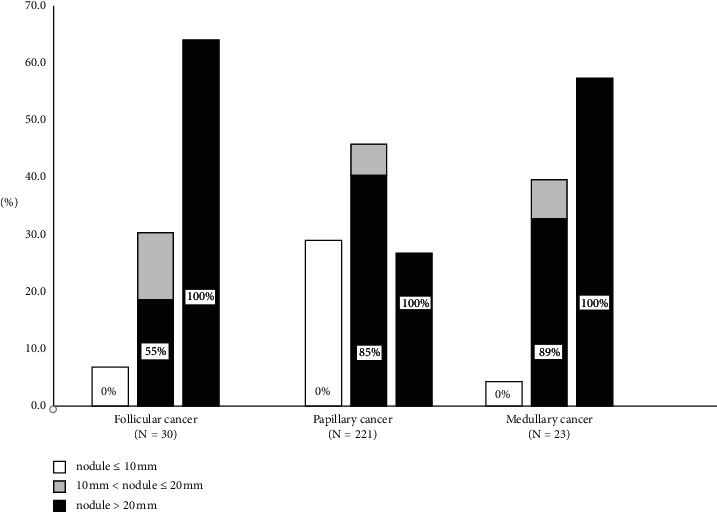
Indication of FNA based on EU-TIRADS. Size distribution of the three major thyroid cancer types; shaded areas show the proportion of the nodules in which the EU-TIRADS-based approach would have indicated FNA. The values in the bars are given in %.

Distribution of cancers according to thyroid nodule size is shown in Figure 1. A larger proportion of PTCs (63/221; 28.5%), compared with non-PTCs (3/53; 5.8%), was ≤10 mm (*p*=0.0005). These carcinomas would have been lost to diagnosis based solely on EU-TIRADS size criteria (Table 2).

A significantly larger proportion of FTCs (19/30; 63.3%) compared with non-FTCs (71/244; 25.9%, *p*=0.0002) was ≥20 mm. In this size range, EU-TIRADS-based decision of FNA would have identified all cancers (Table 2).

**Figure 2:**
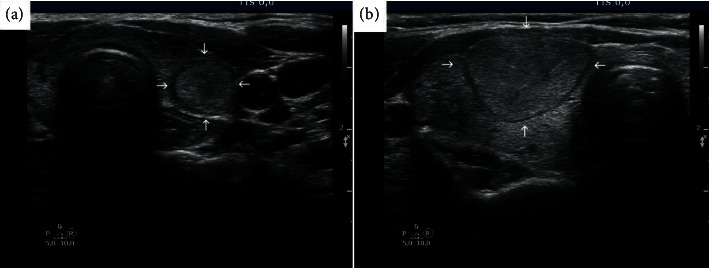
The importance of timely diagnosis of follicular thyroid cancer. (a) An EU-TIRADS score 3 nodule (arrows) with a maximum diameter of 12 mm. According to EU-TIRADS, FNA is not indicated, and the diagnosis would have been missed. Final histology was minimally invasive follicular cancer restricted to the thyroid. (b) A 28 mm follicular cancer (arrows) metastasizing to the lungs. The patient was first examined in another hospital 12 years earlier. At that time, the diameter of the nodule was 14 mm, and FNA was not performed because of the absence of suspicious ultrasound characteristics.

The largest diameter of the nodules was between 10 mm and 20 mm in 118/274 (43.1%) of the carcinomas. This is the subgroup of patients in which the US characteristics influence the decision regarding FNA. EU-TIRADS would have indicated FNA in 88.9% of MTCs, 85.0% of PTCs, and 55.6% of FTCs, the difference between non-FTC and FTC being significant (*p*=0.02) (Table 3). According to the AACE-2006 guideline, we aspirated all nodules, including follicular cancers, in this size range (Figure 2). However, based on US-driven indication of FNA, 44.4% of follicular cancers in the 10–20 mm size range would have escaped detection due to their low EU-TIRADS score (Table 3).

### 2.2. The Distribution of Major Ultrasound Characteristics in the 10–20 mm Size Range

Further analyses were performed in nodules with largest diameter between 10 mm and 20 mm because their US characteristics determine if EU-TIRADS suggests FNA (Table 4). The mean number of the four possible suspicious US features per nodule was lower in FTC (0.67 ± 0.59) compared to MTC (1.78 ± 0.74; *p*=0.01) and PTC (1.68 ± 0.98; *p*=0.002). There was no significant difference between MTC and PTC in this respect (*p*=0.77). The mean EU-TIRADS score was lower in FTC (4.11 ± 0.59) compared with MTC (4.89 ± 0.20) and PTC (4.80 ± 0.33) (*p*=0.02 and *p*=0.01, respectively).

### 2.3. The Clinical Stage in Relation to EU-TIRADS-Based FNA Indication in 10–20 mm Nodules

There were 4 FTCs, 1 MTC, and 15 PTCs among lesions between 10 mm and 20 mm in diameter in which FNA would not have been indicated based on EU-TIRADS alone (Table 5). Two PTC patients presented with a large goiter, which required surgery. Both patients had T1 and stage I thyroid cancer. In the remaining 18 patients, including the 4 FTCs, FNA was the sole diagnostic test directing the patient towards surgery.

Out of these 20 cases, in which FNA would not have been indicated based merely on EU-TIRADS, four were T4 cancers (1 FTC and 3 PTCs) and two were stage IV carcinomas (1 FTC and 1 PTC) in which lung and bone metastases were revealed by postradioiodine therapy SPECT-CT.

## 3. Discussion

Since 2017, EU-TIRADS has been widely used for selection of thyroid nodules for FNA. The EU-TIRADS scores influence the decision of FNA in lesions with a maximum diameter between 10 and 20 mm [8]. In our cohort of patients, FNA would have been indicated in 85% of PTCs, 89% of MTCs, and only 56% of FTCs, if based solely on the EU-TIRADS US criteria. This is a clear underdetection of FTC. For the three cancer types combined, 20 out of 118 cases, including 2 stage IV cancers, would have remained undiagnosed if the recommendations of the EU-TIRADS were followed.

The striking difference in the US-based FNA indication rate between FTC and non-FTC cancers was the consequence of the higher average EU-TIRADS scores in MTC (4.89) and PTC (4.80), compared to FTC (4.11). The substantially higher prevalence of suspicious US characteristics in non-FTC lesions is in accordance with the observations of others [13–16, 21, 22] (Table 4). Furthermore, similar to others, we found no significant difference between PTC and MTC as for the presence of suspicious US features [29, 30] and verified that the performance of EU-TIRADS in the diagnosis of MTC is as good as in PTC [16, 17].

Our findings confirm those of Castellana et al., hitherto the only study focusing on the performance of EU-TIRADS in the diagnosis of FTC [23]. However, while they demonstrated a 6.7% EU-TIRADS failure rate in diagnosing FTCs, it is as high as 20.0% in our study. The explanation lies in the way patients are selected for FNA; in the Castellana et al.' study [23], patients were referred to FNA (and thus surgery) if the US suspicion criteria were fulfilled, i.e., some FTCs have been overlooked due to lack of suspicious signs. The difference in mean tumor diameter of FTCs (33 mm vs. 27 mm in their study and our study, respectively) is in line with this explanation. The larger the proportion of nodules >2 cm in the study population, the lower the rate of failures of EU-TIRADS, because the EU-TIRADS scoring system calls for FNA in <2 cm nodules only if suspicious signs are present. In our study, all nodules larger than 1 cm were sampled, thereby, avoiding selection bias.

Two conflicting views exist regarding the capacity of TIRADS in recognizing FTCs. The guideline of the American Thyroid Association considers it acceptable not to recognize FTCs smaller than 20 mm in their largest diameter because distant metastases occur rarely in such lesions [7]. The main argument in support of this view emphasizes the sparing of FNAs in benign lesions, a fact which has been convincingly shown [3, 11] but is beyond the scope of the current study. The ATA view is further supported by the lower incidence of FTC in areas of long ago achieved iodine sufficiency, such as the United States. The other view states that one has to exert every effort to recognize FTCs well before they reach 20 mm in diameter, because once the nodule becomes larger, distant metastases may evolve [31]. Furthermore, in Europe, FTC incidence remains around 10% of all thyroid cancers [32].

Compared to PTCs, FTCs are more likely to be iso or hyperechoic, noncalcified, round shaped, and halo encompassed with regular margins [33]. Conceivably, US risk stratification is fundamentally inadequate in identifying nodules that could be FTCs. One should be aware that while chasing PTCs, we are, to some extent, neglecting a more aggressive type of thyroid cancer [24, 27]. Furthermore, this eliminates the fundamental problem that FTCs can only be distinguished from follicular adenomas by histology. Considering the 1 : 16 to 1 : 5 ratio of FTC to follicular adenoma [34, 35], a large number of unnecessary FNAs could be spared if one were less committed to recognize FTC than PTC. The reliability of the current approaches in detecting FTCs should still be improved [27], either by modifying patterns and cutoffs for FNA or by integrating US with other technologies. Once selected for FNA, molecular testing and nuclear techniques [36] are future candidates for discriminating between follicular adenomas and follicular cancers.

As a main strength of the present study, we have tested the diagnostic sensitivity of an EU-TIRADS system in a cohort selected for FNA and surgery based on the pre-TIRADS approach, rather than the current focus on limiting number of FNAs. The true sensitivity of a method, in this case, TIRADS, can be tested only in such a nonrestricted cohort of patients. Most, if not all, recently published studies have estimated the diagnostic performance of TIRADS in cohorts selected for FNA and surgery based on the same principles on which the tested TIRADS method is based [3, 10, 11, 14, 37, 38]. This implies that nodules which failed to show the required features of high suspicion escaped FNA and surgery and were therefore not diagnosed as malignant. This selection bias has prevented recognition of the low sensitivity of EU-TIRADS in detecting FTCs in the 10–20 mm range.

Our study design focused exclusively on the sensitivity, not the specificity, of EU-TIRADS. We acknowledge the merits of EU-TIRADS in the reduction of superfluous FNAs. This unquestionable benefit could have been investigated in patients operated on for benign lesions. However, in the present study, in almost all such patients, the dominant nodule was larger than 2 cm, where TIRADS, independently of the score, suggests FNA.

The limitations of our study are its retrospective nature and the limited number of thyroid cancers in the relevant size range. Inherent inaccuracy of the cytology evaluation may have distorted the final results. Furthermore, this has been a single center study with all of the drawbacks of such observations, including limitations in generalizability. This limitation is even more important, in that all examinations were performed by the same investigator. Thus, our findings are calling for confirmation.

In conclusion, when evaluating the performance of EU-TIRADS, analyses should focus preferentially on lesions in the size range where the US characteristics have a real impact on the indication for FNA, the 10–20 mm range. In this range, EU-TIRADS identified the majority of patients to be sampled for cytology in PTCs and MTCs, but indicated FNA only in half of the FTCs. TIRADS-based US criteria favor PTC and MTC characteristics over that of FTC. The diagnostic performance of TIRADS cannot be established in cohorts of patients in which the selection for FNA and surgery is based on suspicion criteria in which the TIRADS is based on. Such an approach underestimates the false-negative FTC rate of TIRADS-based decisions. Prospective studies to clarify the exact proportion of FTCs missed are warranted.

## Figures and Tables

**Table 1:** The clinical and histopathological data of the 274 thyroid cancer patients.

	Follicular cancer (*N* = 30)	Papillary cancer (*N* = 221)	Medullary cancer (*N* = 23)
Male : female	5 : 25	45 : 176	5 : 18

Age (years) mean ± SD	50.4 ± 17.1	43.4 ± 15.0	56.6 ± 17.0

Maximum diameter of the tumor (mm), mean ± SD	27.2 ± 12.4	17.3 ± 9.9	26.2 ± 17.0

Results of FNA according to the Bethesda classification^1^	1: 1	1: 14	1: 0
	2: 1	2: 8	2: 1
	3: 1	3: 1	3: 0
	4: 22	4: 13	4: 0
	5: 5	5: 68	5: 5
	6: 0	6: 117	6: 17

Histopathological stage^1^	Stage I: 11	Stage I: 139	Stage I: 7
	Stage II: 11	Stage II: 46	Stage II: 6
	Stage III: 3	Stage III: 28	Stage III: 6
	Stage IV: 5	Stage IV: 8	Stage IV: 4

^1^Histopathological staging according to reference [28].

**Table 2:** Evaluation of nodular goiter based on the 2006 Guideline of the AACE/ACE and on the EU-TIRADS in relation to missing the cancer diagnosis in three subtypes of thyroid cancers.

Maximal diameter of the nodule	Indication of FNA		
	≤10 mm	10 mm–20 mm	>20 mm
AACE/ACE guideline (2006)	Suspicious lesion	All nodules	All nodules
EU-TIRADS (2017)	If equivocal^1^	Depending on the EU-TIRADS score	All nodules^2^
Overlooked carcinomas by EU-TIRADS (% of all cancers of that type irrespective of nodule size)	6.7% of FTC^3^	13.3% of FTC	None
	4.3% of MTC^3^	4.3% of MTC	None
	28.5% of PTC^3^	5.2% of PTC	None
Overlooked carcinomas by EU-TIRADS (% of cancers within the specific size limits)	100% of FTC^3^	44.4% of FTC	None
	100% of MTC^3^	11.1% of MTC	None
	100% of PTC^3^	15.0% of PTC	None
Inaccuracy of FNA: nondiagnostic and false-negative results (related to all cancers of that type)	3.3% of FTC	0% in FTC	3.3% of FTC
	0% of MTC	4.3% of MTC	0% in MTC
	1.8% of PTC	3.2% of PTC	5.0% of PTC

EU-TIRADS, Thyroid Imaging Reporting and Data System of the European Thyroid Association [8]; AACE/ACE, American Association of Clinical Endocrinologists (AACE) and American College of Endocrinology (ACE) [2]. ^1^EU-TIRADS suggests “FNA or active surveillance.” ^2^Except for pure cysts and entirely spongiform cysts. ^3^Overlooked if FNA would not have been performed in suspicious lesions ≤10 mm.

**Table 3:** EU-TIRADS-based indication of FNA. Both the EU-TIRADS score and nodule size influence the indication.

Largest diameter	Follicular cancer (*n* = 30)		Medullary cancer (*n* = 23)		Papillary cancer (*n* = 221)	
	Number of lesions	FNA indicated, *n* (%)	Number of lesions	FNA indicated, *n* (%)	Number of lesions	FNA indicated^1^, *n* (%)
≤10 mm	2	0 (0)	1	0 (0)	63	0 (0)
10–20 mm	9	5 (55.6)	9	8 (88.9)	100	85 (85.0)
>20 mm	19	19 (100)	13	13 (100)	58	58 (100)

EU-TIRADS, Thyroid Imaging Reporting and Data System of the European Thyroid Association. ^1^According to the suggestions of the EU-TIRADS, FNA was considered indicated even if it was substantiated only on the presence of pathological lymph nodes.

**Table 4:** The occurrence of individual suspicious ultrasound characteristics in three subtypes of thyroid cancer. For comparison, previously published data are given ([11], [14–17], [22], [33], [39–56]).

	Follicular cancer		Medullary cancer		Papillary cancer	
	Mean ± SD	Median (range)	Mean ± SD	Median (range)	Mean ± SD	Median (range)
Marked hypoechogenicity						
Present study	22.2 ± 19.4		44.4 ± 27.8		25.0 ± 43.5	
Literature data	**5.2** **±** **15.3**	3.6 (2.5–10.9)	**29.3** **±** **11.1**	32.7 (19.6–52.4)	**38.5** **±** **24.6**	32.7 (17.1-84-9)
Microcalcifications						
Present study	44.4 ± 27.8		44.4 ± 27.8		65.0 ± 47.9	
Literature data	**16.2** **±** **22.1**	7.7 (0–61.5)	37.0 ± 8.8	37.5 (16.7–47.2)	**49.5** **±** **25.0**	50.9 (17.8–89.1)
Irregular shape						
Present study	11.1 ± 11.1		33.3 ± 50.0		32.0 ± 22.0	
Literature data	**17.0** **±** **17.5**	14.1 (0–44.4)	27.4 ± 19.7	36.4 (2.9–63.1)	**40.8** **±** **25.0**	36.4 (9.6–74.0)
Irregular borders						
Present study	33.3 ± 50.0		55.6 ± 52.7		46.0 ± 50.0	
Literature data	**23.3** **±** **19.4**	22.0 (0–60.9)	**37.9** **±** **22.4**	45.2 (0–63.6)	**48.2** **±** **28.9**	40.4 (22.3–92.9)

All values are given in %.

**Table 5:** EU-TIRADS score-based indication of FNA related to tumor stage in the 10–20 mm size category.

Cancer type	*n*	TNM status		Pathological staging			
		T1	T2–T4	Stage I	Stage II	Stage III	Stage IV
Papillary (*n* = 100)							
FNA indicated	85	68	17	76	7	1	1
FNA not indicated	15	12	3	12	2	0	1
Follicular (*n* = 9)							
FNA indicated	5	5	0	5	0	0	0
FNA not indicated	4	3	1	3	0	0	1
Medullary (*n* = 9)							
FNA indicated	8	5	34	0	4	0	
FNA not indicated	1	1	0	1	0	0	0
All (*n* = 118)							
FNA indicated	98	75	23	73	8	1	16
FNA not indicated	20	16	4	16	2	0	2

EU-TIRADS, Thyroid Imaging Reporting and Data System of the European Thyroid Association [8].

## Data Availability

The data used to support the findings of this study are included within the article.

## Additional Points

EU-TIRADS does not recognize half of the follicular cancers in the 10–20 mm range. Virtually, all studies in this field share a selection bias: the sensitivity of EU-TIRADS is tested in cohorts selected for cytology/surgery based on EU-TIRADS ultrasound suspicion criteria.

## Supplementary Materials

Supplementary Materials Supplementary Figure 1. The occurrence of individual suspicious ultrasound characteristics in three subtypes of thyroid cancer. For comparison, previously published data are shown (refs [11], [14–17], [22], [33], [39–56]). FTC – follicular thyroid cancer, MTC – medullary thyroid cancer, PTC – papillary thyroid cancer. All values are %.
