# Supplementary material for: EU-TIRADS-Based Omission of Fine-Needle Aspiration and Cytology from Thyroid Nodules Overlooks a Substantial Number of Follicular Thyroid Cancers
Source: Int J Endocrinol. 2021 Sep 27;2021:9924041. doi: 10.1155/2021/9924041 (PMC8490077; doi:10.1155/2021/9924041)

**Supplementary Figure 1.** The occurrence of individual suspicious ultrasound characteristics in three subtypes of thyroid cancer. For comparison, previously published data are shown (refs **11, 14-17, 22, 33, 39-56**). FTC – follicular thyroid cancer, MTC – medullary thyroid cancer, PTC – papillary thyroid cancer. All values are %.


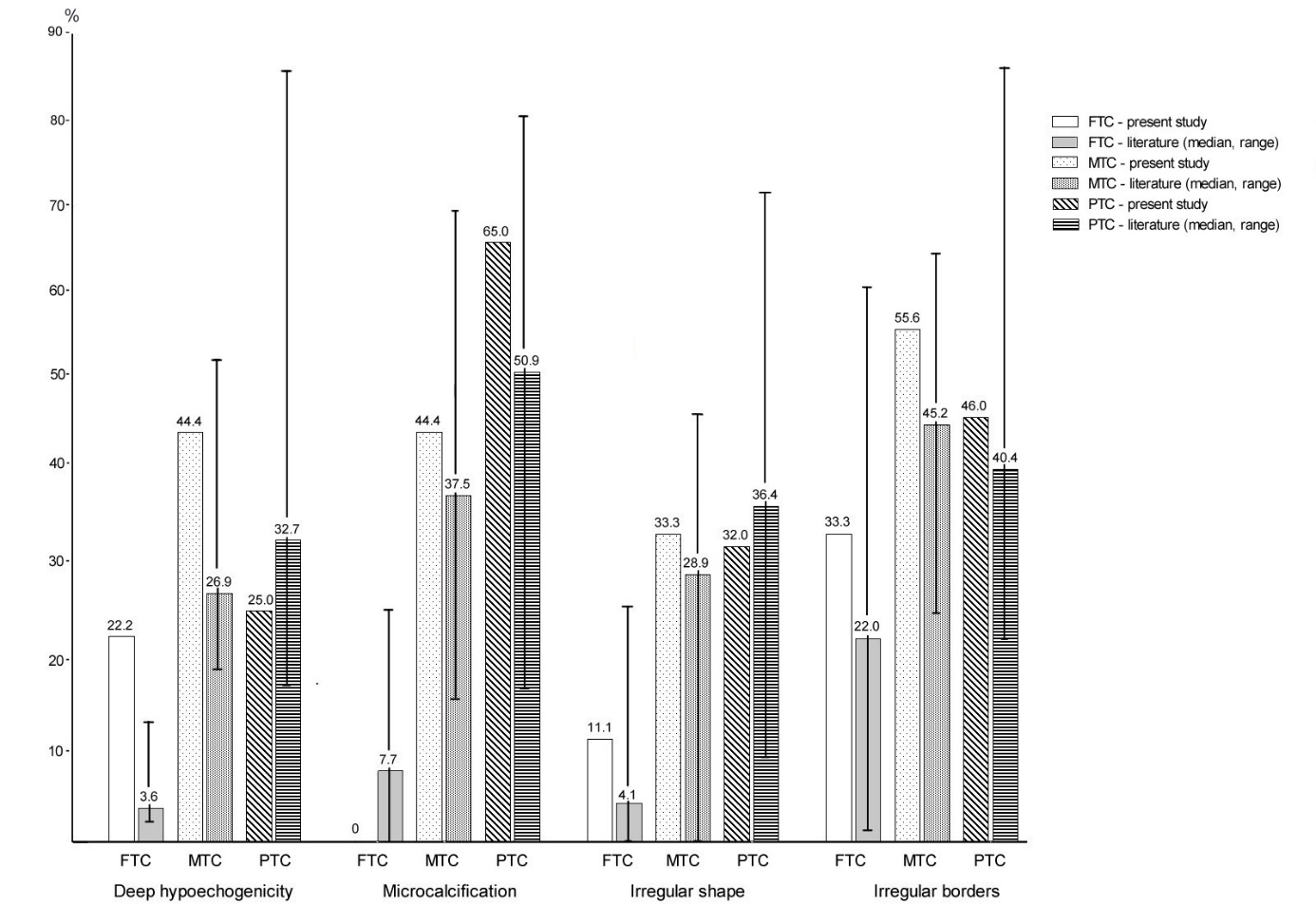

Supplement: Supplementary Materials — Supplementary Figure 1. The occurrence of individual suspicious ultrasound characteristics in three subtypes of thyroid cancer. For comparison, previously published data are shown (refs [11], [14–17], [22], [33], [39–56]). FTC – follicular thyroid cancer, MTC – medullary thyroid cancer, PTC – papillary thyroid cancer. All values are %. [file 9924041.f1.docx]
